# Exploring Spatial and Temporal Distribution of Cutaneous Leishmaniasis in the Americas, 2001–2011

**DOI:** 10.1371/journal.pntd.0005086

**Published:** 2016-11-08

**Authors:** Ana Nilce Silveira Maia-Elkhoury, Zaida E. Yadón, Martha Idali Saboyá Díaz, Francisca de Fátima de Araújo Lucena, Luis Gerardo Castellanos, Manuel J. Sanchez-Vazquez

**Affiliations:** 1 Communicable Diseases and Health Analysis, Pan American Health Organization, Duque de Caxias, Rio de Janeiro, Brazil; 2 Communicable Diseases and Health Analysis, Pan American Health Organization, Washington, District of Columbia, United States of America; 3 Ministry of Social Development and Against Hungry, Brasilia, Federal District, Brazil; 4 Pan American Foot-and-Mouth Disease Center–PANAFTOSA/PAHO, Duque de Caxias, Rio de Janeiro, Brazil; Institut de Recherche pour le Développement, FRANCE

## Abstract

Leishmaniasis is an important health problem in several countries in the Americas and cases notification is limited and underreported. In 2008, the Pan American Health Organization (PAHO/WHO) met with endemic countries to discuss the status and need of improvement of systems region-wide. The objective is to describe the temporal and spatial distribution of cutaneous leishmaniasis (CL) cases reported to PAHO/WHO by the endemic countries between 2001 and 2011 in the Americas.

**Methods:**

Cases reported in the period of 2001–2011 from 14/18 CL endemic countries were included in this study by using two spreadsheet to collect the data. Two indicators were analyzed: CL cases and incidence rate. The local regression method was used to analyze case trends and incidence rates for all the studied period, and for 2011 the spatial distribution of each indicator was analyzed by quartile and stratified into four groups.

**Results:**

From 2001–2011, 636,683 CL cases were reported by 14 countries and with an increase of 30% of the reported cases. The average incidence rate in the Americas was 15.89/100,000 inhabitants. In 2011, 15 countries reported cases in 180 from a total of 292 units of first subnational level. The global incidence rate for all countries was 17.42 cases per 100,000 inhabitants; while in 180 administrative units at the first subnational level, the average incidence rate was 57.52/100,000 inhabitants. Nicaragua and Panama had the highest incidence but more cases occurred in Brazil and Colombia. Spatial distribution was heterogeneous for each indicator, and when analyzed in different administrative level. The results showed different distribution patterns, illustrating the limitation of the use of individual indicators and the need to classify higher-risk areas in order to prioritize the actions. This study shows the epidemiological patterns using secondary data and the importance of using multiple indicators to define and characterize smaller territorial units for surveillance and control of leishmaniasis.

## Author Summary

In the Americas, cutaneous leishmaniasis (CL) cases are notified across a wide geographic area, extending from southern United States to northern Argentina. Currently, 70–75% of all estimated cases of CL worldwide occur in ten countries, including four in the Americas (Brazil, Colombia, Nicaragua, and Peru) and six countries from Africa and the Eastern Mediterranean. This study shows the epidemiological situation and geographical distribution of CL cases reported by passive surveillance system of endemic countries of the American Region from 2001 to 2011, collected and consolidated by the Pan American Health Organization (PAHO). Furthermore, it represents a joint effort of the National Programs of leishmaniases. Despite some limitations, the data were analyzed and discussed and this study represents the first step to understand the global epidemiological situation in this Region and it contributes to the improvement of the surveillance of leishmaniases in the Americas.

## Introduction

Leishmaniasis is an important health problem for several countries in the Americas. It is caused by different species of protozoans of the genus *Leishmania* and transmitted to humans and animals by insect vectors of the Psycodidae family infected during blood feeding on vertebrate reservoirs and hosts [1–3]. Various factors including climate, economic, environmental, social, and others, have an influence in the leishmaniasis transmission cycle and thus determining the epidemiological patterns of the disease [3–5].

The disease occurs in different clinical forms (cutaneous, mucosal, and visceral), according to the parasite. Aspects such as the immunogenetic profile of the affected population, malnutrition, and access to health systems, among others, have an influence in the prognosis of the disease, which can progress from mild to severe, causing deformities, mutilation, and even death [3, 5].

A recent study shows that leishmaniasis had an increase of 8.8% (-6.4 to 25.3%) in the disability-adjusted life year (DALYs) for all ages when comparing 2013 to 2005, and a significantly higher increment for the cutaneous and mucocutaneous forms (35.9%–23.7 to 49.0%) [6].

Globally, Cutaneous leishmaniasis (CL) is broadly distributed occurring in 83 countries, with an estimated of 0.7–1.2 million new cases per year [7]. Currently, 70–75% of all estimated cases of CL worldwide occur in ten countries: four in the Americas (Brazil, Colombia, Nicaragua, and Peru) and six in Africa and Eastern Mediterranean (Afghanistan, Algeria, Iran, Syria, Ethiopia, and Sudan) [3, 7]. In the Americas, CL cases have been notified across a wide geographic area extending from southern United States to northern Argentina [3].

In the past decades it has become apparent that CL is more prevalent in many areas of the Latin America than previously thought. This uncertainty about its occurrence is partially because the notification in many endemic countries was often underreported and the collected information remained limited [7, 8]. A comprehensive understanding of the transmission of leishmaniasis, incorporating the knowledge of specific spatial and temporal distribution of the disease, is essential to make decisions in order to direct and prioritize actions for surveillance and control strategies for cutaneous leishmaniasis [9]. At this juncture, the Pan American Health Organization/World Health Organization (PAHO/WHO) Regional Leishmaniasis Program, brought together (in Colombia 2008), the coordinators of the National Leishmaniasis Control Programs of the American Region to discuss the status of the leishmaniasis reporting system and agreed upon the development of a regional information and surveillance systems region-wide coordinated by PAHO/WHO. The meeting helped jumpstart the debate and definition of criteria for an improved epidemiological surveillance system to fully understand the epidemiological status of leishmaniasis in the Americas.

The objective of this study is to describe the temporal and spatial distribution of CL cases between 2001 and 2011 utilizing the data reported to PAHO/WHO by the endemic countries of the Americas, as a first step to determine the time and spatial distribution of the disease in the subregions and region.

## Materials and Methods

### Data collection

Over 2011 and 2012, the Regional Leishmaniasis Program of PAHO/WHO coordinated the data collection among endemic countries, requesting the annual data of CL from 2001 to 2011 through two spreadsheet templates. One spreadsheet to collect national data standardized by country, year, and clinical form of the disease for the whole study period; and a second spreadsheet to collect only data from 2011 disaggregated at the first subnational administrative level (department, state, or province, depending on the country). Additionally, consolidated, aggregated, and analyzed all the information provided by the endemic countries using the regional database [10].

There were many differences between countries in their leishmaniasis surveillance systems, including the process of reporting. Thus, while case notification for leishmaniasis is mandatory region-wide, reporting can be done on individual or aggregate basis, depending on the specific criteria established by the national epidemiological surveillance system. The definition of cases, notification process, and variables for each data collected were also different among the countries due to the different clinical forms, internal system and information flow established in each country. This scenario of uneven data structure defined the limitations of the potential analyses to be carried out in this study.

The data collection included cases reported from 2001–2011 from 14 of the 18 endemic American countries (Argentina, Brazil, Bolivia, Colombia, Costa Rica, El Salvador, Ecuador, Guatemala, Guyana, Honduras, Nicaragua, Panama, Paraguay, and Peru). French Guiana, Mexico, Suriname, and Venezuela, did not complete the spreadsheets templates provided by PAHO/WHO to report data in the time requested. For the 2011 spatial analysis, 15 countries participated (the 14 countries listed above plus Mexico) and provided information on the following variables: total number of CL cases, total population of the administrative unit (national or first subnational administrative department, state, or province) where the cases occurred.

### Data analysis

#### CL case and incidence rate

In order to analyze and present basic health indicators [11], PAHO/WHO divides the American Region into six subregions: 1) Andean area (Bolivia, Colombia, Ecuador, and Peru); 2) Central America (Costa Rica, El Salvador, Guatemala, Honduras, Nicaragua, and Panama); 3) Brazil; 4) Southern Cone (Argentina and Paraguay); 5) the non-Latin Caribbean (Guyana); and 6) Mexico. The same subregions were used for data analysis in this study.

Fourteen countries were analyzed for the period of 2001–2001 for two indicators: CL cases and CL incidence. The incidence rate was calculated by dividing the number of confirmed cases by the country population at each year-end and multiplied by 100,000. The population data were obtained from the official census of each country. In 2011, the incidence rate was calculated at the national and first subnational administrative level using the method described above.

Microsoft Office Excel 2007 (Microsoft Corporation, Redmond, Washington, United States) was used to compile and analyze the data.

#### Temporal distribution of CL cases and incidence rates

The temporal distribution was evaluated at three levels of geographical units: i.e. region, subregion and country. The local regression method, known by the acronym LOESS (locally weighted scatterplot smoothing) [12, 13], was used to identify the trend line for the number of cases and incidence. LOESS is an exploratory statistical method that allows a least-squares regression curve to be represented through a series of data points which defines two parameters: 1) the value of the smoothing parameter that defines the subset of data used (either the nearest or the furthest), expressed as “α” and 2) the degree of the local polynomial. To adjust the curve, several combinations of parameters were attempted, minimizing an excessive weight in the smoothing (to avoid over-adjustment). Based on the results of these attempts, an **α** value of 0.66 was utilized with a linear weighting (a first-degree local polynomial). Graphs depicting the data for the subregions, were represented with data points and the fitted trend line. The analyses were conducted with the R statistical software environment [14], specifically the “stats” library.

#### Spatial distribution of CL cases and incidence rates for 2011

For the spatial analysis, the number of cases and incidence rates for CL reported in 2011 were linked to the national and first subnational administrative data represented by polygons. Subsequently, the data were categorized in quartiles, which allowed distributing the values of each indicator into four groups containing equal number of units. These categories were utilized to develop choropleth maps (scaling the color according to the value) with the distribution of the two CL indicators, both at the national and first subnational administrative levels across the Region.

The maps were generated with the ArcGIS program (ESRI, Redlands, California, United States). The administrative boundaries at the country level were obtained from the Global Administrative Areas Project (http://gadm.org/) and the boundaries of the first subnational administrative level were obtained from the Second Administrative Level Boundaries data set (SALB).

### Ethical Considerations

In this study the legal principles and ethical aspects were considered and respected. These data refer to the routine of a control program of leishmaniasis and surveillance services of the Health Ministries of endemic countries, which maintained confidential the identification of patients.

## Results

### CL cases: 2001–2011 period

In the period of 2001–2011, 14 countries reported a total of 636,683 CL cases, with an annual average of 57,923 cases (ranging from 47,286 to 67,949). A total of 270,572 of the reported cases (42.5%) were from Brazil; 256,261 (40.2%) were from countries of the Andean subregion; 100,475 (15.8%) were from Central American countries; and 9,375 (1.5%) were reported by Argentina, Guyana, and Paraguay, Table 1.

**Table 1:** Number of cases and incidence (Incid) rate of cutaneous leishmaniasis per 100.000 inhabitants, reported per year for the 14 selected endemic countries and by subregion, Americas region, 2001–2011. **Source**: PAHO/WHO. Data reported by the leishmaniasis control programs of the countries to PAHO/WHO through May 2012.

Sub-region/ Countries	2001		2002		2003		2004		2005		2006		2007		2008		2009		2010		2011	
	Cases	Incid	Cases	Incid	Cases	Incid	Cases	Incid	Cases	Incid	Cases	Incid	Cases	Incid	Cases	Incid	Cases	Incid	Cases	Incid	Cases	Incid
**Americas**	**47286**	**13.89**	**56243**	**16.26**	**61518**	**17.55**	**59439**	**16.76**	**67949**	**18.78**	**62017**	**16.90**	**59027**	**15.88**	**52324**	**13.97**	**57265**	**15.10**	**58347**	**15.33**	**53823**	**13.96**
***Andean***	***13165***	***14*.*96***	***17841***	***19*.*74***	***19864***	***21*.*67***	***22860***	***24*.*79***	***30697***	***32*.*86***	***29177***	***30*.*84***	***27852***	***29*.*06***	***20562***	***21*.*19***	***24886***	***25*.*22***	***25868***	***25*.*97***	***22132***	***21*.*88***
Bolivia	2043	23.57	2518	28.47	2452	27.19	2819	30.68	2657	28.40	3152	33.12	3153	32.58	1838	18.69	1218	12.19	1809	17.81	1636	15.85
Colombia	4130	10.12	7038	17.03	9267	22.14	10698	25.25	18043	42.07	16241	37.42	13331	30.35	9595	21.59	15420	34.28	14818	32.56	9228	20.04
Ecuador	1754	14.43	1253	9.32	1336	9.74	2494	18.88	1925	14.40	1536	11.34	1185	8.61	1479	10.62	1735	11.91	1629	11.25	965	6.43
Peru	5238	19.87	7032	26.30	6809	25.12	6849	24.94	8072	29.02	8248	29.30	10183	35.75	7650	26.56	6513	22.36	7612	25.84	10303	34.58
***Central America***	***7186***	***19*.*74***	***8135***	***21*.*90***	***9336***	***24*.*73***	***6744***	***17*.*51***	***9687***	***24*.*84***	***9717***	***24*.*45***	***8903***	***22*.*04***	***11037***	***26*.*84***	***9959***	***23*.*79***	***9637***	***22*.*62***	***9994***	***23*.*05***
Costa Rica	425	10.75	690	17.15	948	23.20	1061	25.56	1676	39.76	1870	43.71	1807	41.63	818	18.57	2025	45.31	1143	25.21	1376	29.96
El Salvador	18	0.30	46	0.77	24	0.40	76	1.26	24	0.40	46	0.76	36	0.59	31	0.51	-*	-*	4	0.06	17	0.27
Guatemala	-*	-*	1549	13.14	1143	9.46	870	7.02	1243	9.79	602	4.62	287	2.15	494	3.61	519	3.70	410	2.85	549	3.73
Honduras	957	14.65	1260	18.82	1684	24.55	797	11.34	1574	21.87	1300	17.65	855	11.34	1759	22.82	1502	19.07	1362	16.93	1685	20.51
Nicaragua	2924	54.74	2200	40.13	3716	67.78	2103	37.38	3521	64.21	2125	37.99	3719	66.37	5826	102.77	4047	70.48	3497	60.13	3146	53.42
Panama	2862	92.26	2390	75.53	1821	56.44	1837	55.86	1649	49.21	3774	110.56	2199	63.27	2109	59.61	1866	51.83	3221	87.96	3221	86.50
***Brazil***	***26328***	***15*.*27***	***28268***	***16*.*19***	***30812***	***17*.*42***	***28737***	***16*.*04***	***26685***	***14*.*49***	***22397***	***11*.*99***	***21530***	***11*.*37***	***20123***	***10*.*61***	***21989***	***11*.*48***	***22397***	***11*.*74***	***21356***	***11*.*10***
***Southern Cone***	***607***	***1*.*42***	***1999***	***4*.*64***	***1496***	***3*.*44***	***1089***	***2*.*47***	***873***	***1*.*96***	***720***	***1*.*60***	***736***	***1*.*62***	***588***	***1*.*28***	***422***	***0*.*91***	***430***	***0*.*92***	***326***	***0*.*68***
Argentina	157	0.42	748	1.99	348	0.92	358	0.94	282	0.73	257	0.66	201	0.51	208	0.52	163	0.41	166	0.41	140	0.34
Paraguay	450	7.99	1251	22.51	1148	20.22	731	12.63	591	10.02	463	7.70	535	8.74	380	6.10	259	4.08	264	4.09	186	2.83
***No-Latin Caribean***	-*	-*	-*	-*	***10***	***1*.*33***	***9***	***1*.*19***	***7***	***0*.*92***	***6***	***0*.*78***	***6***	***0*.*78***	***14***	***1*.*80***	***9***	***1*.*15***	***15***	***1*.*91***	***15***	***1*.*90***
Guyana	-*	-*	-*	-*	10	1.33	9	1.19	7	0.92	6	0.78	6	0.78	14	1.80	9	1.15	15	1.91	15	1.90

*-: cases not recorded in the year.

**Fig 1:**
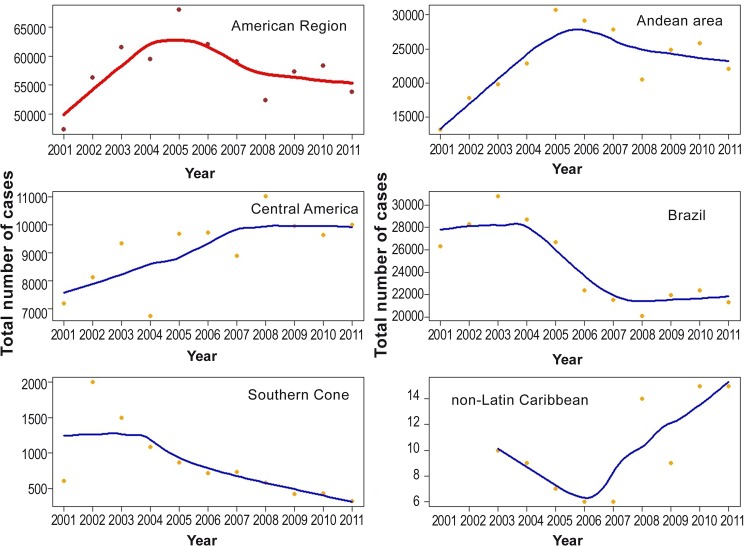
Number of cutaneous leishmaniasis cases reported by 14 endemic countries (overall), and stratified by sub region, Americas region, 2001–2011. **The Plotted points correspond to actual counts of cases. The Lines consist in short term trends (fitted with LOESS).** Source: PAHO/WHO. Data reported by the leishmaniasis control programs of the countries to PAHO/WHO through May 2012.

In the first half of the studied period (2001–2005) there was a continuous increase of the total number of CL cases reported per year, which increased 44% in 2005 when compared to 2001. In the second half of the period (2006–2011) there was a decline of 11% of the CL in this region, but resulting in an overall increase of 30% for all study period, Table 1, Fig 1.

The number of cases reported per year by subregion for the period of 2001–2011, showed an overall increase of 68% and 39% in the Andean countries and in Central America, respectively. For the same period, in both Brazil and the Southern Cone there was a decline of the total number of cases. However, in the Southern Cone the number of CL cases reported tripled between 2001 and 2002, Fig 1.

Two countries in the Andean subregion (Colombia and Peru), reported an increase of cases during the overall studied period. In Colombia, the reported cases increased 4.36-fold in 2005 versus 2001. In Peru, there was a continuous increase of cases over the period, with the number of cases increasing 2.13-fold (Table 1). Of the total cases reported between 2001 and 2011 from the Andean subregion, Colombia accounted for 50% of the cases, followed by Peru with 33%, except for 2011, when Peru reported 47.7% of the total CL of the subregions.

In the Central America subregion, the countries that contributed to the overall increase in the occurrence of CL for this period were: Costa Rica (4.7 times higher); Honduras (1.8 times higher); Panama (1.3 times higher) and Nicaragua (2.0 times higher) (Table 1). In the cumulative total of cases for the studied period, Nicaragua reported 36.74%, followed by Panama, which reported 26.82%. This situation was similar every year except in 2006, when Panama reported more cases than Nicaragua, Table 1.

### CL incidence rate: period of 2001–2011

**Fig 2:**
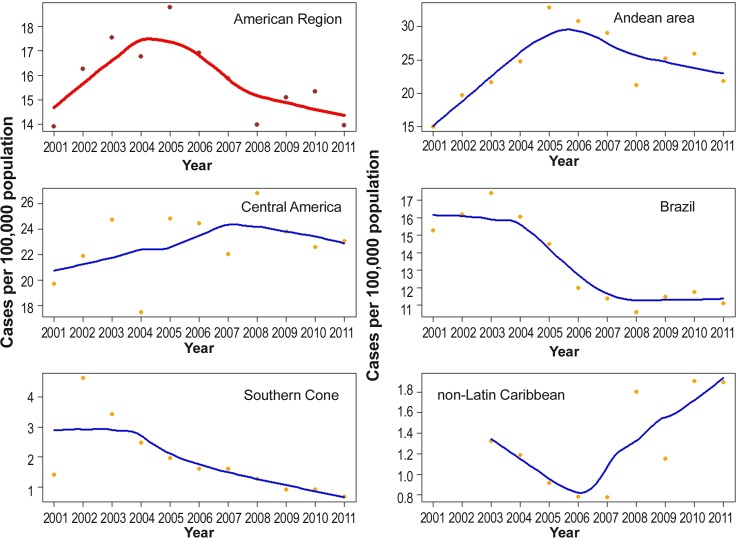
Annual incidence of cutaneous leishmaniasis per 100,000 inhabitants reported by 14 endemic countries (overall), and stratified by sub region, Americas region, 2001–2011. **The Plotted points correspond to actual CL incidences. The Lines consist in short term trends (fitted with LOESS).** Source: PAHO/WHO. Data reported by the leishmaniasis control programs of the countries to PAHO/WHO through May 2012.

The annual CL incidence for the 14 selected endemic countries collectively and by subregion is shown in Table 1. The average incidence of CL was 15.89 cases per 100,000 inhabitants. The incidence increased 35% in the first period (2001 to 2005), but in the second half (2006 to 2011) there was a 21% reduction (Fig 2). In the Andean and Central America subregions, the average incidence rates were higher than the average for all 14 countries, with an incidence of 24.50 and 23.70 cases per 100,000 inhabitants respectively.

Colombia and Peru showed the highest incidence rates for the Andean subregion (Table 1). On the other hand, the incidence rate in the Southern Cone declined (in both Argentina and Paraguay), Table 1.

Central America countries presented the highest CL incidence rates during the period, and among these Panama had the highest number, followed by Nicaragua and Costa Rica, which had higher incidence in 2009 (Table 1).

### Spatial distribution of CL cases and incidence rates for 2011

Within the 15 analyzed countries, 180 (61.6%) out of 292 administrative units at the first subnational level (departments, states, or provinces) reported cases of CL, with an average of 309.64 cases (ranging from 1 to 3,667). Eight countries (Bolivia, Brazil, Colombia, Costa Rica, Nicaragua, Panama, Paraguay, and Peru) reported cases in more than 75% of their first-subnational-level units. The overall incidence rate for all 15 countries in 2011 was 17.42 cases per 100,000 inhabitants, while for the 180 affected administrative units at the first subnational level, the average incidence rate was 57.52 per 100,000 inhabitants (ranging from 0.03 to 1,229.01).

#### Stratification at the national level by quartile

**Fig 3:**
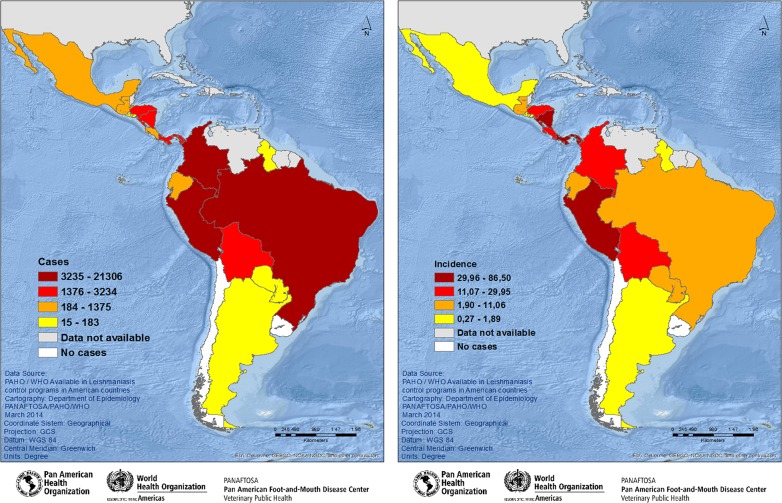
**Spatial distribution of cutaneous leishmaniasis cases (A) and incidence rates per 100,000 inhabitants (B), stratified at national level by quartile, Americas region, 2011.** (3A) Group 1- countries with ≥ 3 235 cases; Group 2—>1 376 to ≤ 3 234 cases; Group 3—>184 to ≤ 1 375 cases; and Group 4—≤183 cases. (3B) Group 1- Incidence rate ≥ 29.96/100 000 inhabitants; Group 2—incidence rate >11.07 to ≤ 29.95 cases/100 000 inhabitants; Group 3—incidence rate >1.90 ≤ 11.06 cases/100 000 inhabitants; and Group 4—≤ 1.989 cases/100 000 inhabitants. Source: PAHO/WHO. Data reported by the leishmaniasis control programs of the countries to PAHO/WHO through May 2012.

The 2011 results at national level were distributed by quartile, and the 15 countries were stratified into four groups for each indicator, Fig 3A and 3B. The distribution of the countries by quartile was heterogeneous for each indicator. Thus, for case distribution, Brazil, Colombia, and Peru were in the top quartile (with totals ranging from 3,235 to 21,306 cases), whereas for incidence rate distribution, Nicaragua, Panama, and Peru were in the top quartile (with totals ranging from 29.96 to 86.50 cases per 100,000 inhabitants).

#### Stratification at the first subnational administrative level by quartile

**Fig 4:**
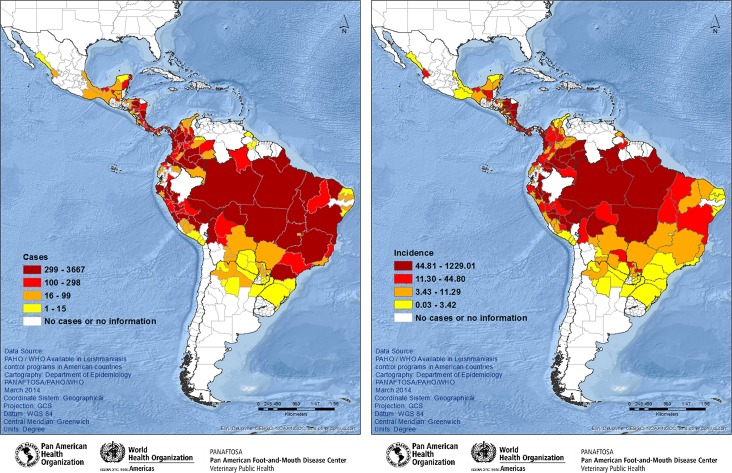
**Spatial distribution of cutaneous leishmaniasis stratified by quartile for cases (A) and incidence rate of cases/100,000 inhabitants (B) at the first subnational administrative level, Americas region, 2011.** (4A) Group 1—Units at the first subnational level with ≥299; Group 2—> 100 to ≤ 298 cases; Group 3—> 16 to ≤ 99 cases; and Group 4—≤ cases. (4B) Group 1- Units at the subnational administrative level with incidence rates ≥44.81 per 100 000 inhabitants; Group 2—incidence rates > 11.30 to ≤44.80 cases per 100 000 inhabitants; Group 3—incidence rates > 3.43 ≤ 11.29 cases per 100 000 inhabitants; and Group 4—incidence rates ≤ 3.43 cases per 100 000 inhabitants. Source: PAHO/WHO. Data reported by the leishmaniasis control programs of the countries to PAHO/WHO through May 2012.

The data for each indicator on case distribution and incidence rates at the first subnational administrative level for 2011 were stratified by quartile for each indicator, and by administrative units at the first subnational level, Fig 4A and 4B.

In 2011, spatial distribution by quartile was also heterogeneous for each of the two indicators in the investigation at the first subnational administrative. For example, Guainía in Colombia, Amazonas in Brazil, and Beni in Bolivia were in the top quartile for incidence rates (44.81 to 1,229 cases per 100,000 inhabitants). Paraná in Brazil and Lima in Peru were in the top quartile for case distribution (299 to 3,667 cases), but in the bottom quartile for incidence (0.03 to 3.42 and 3.43 to 11.29 cases per 100,000 inhabitants respectively).

## Discussion

For the first time the temporal and geographical distribution of CL cases is described in the American Region. The study find that from 2001 to 2011 a total of 636,683 CL cases were reported by the passive surveillance system from 14 endemic countries representing a 30% increase in the period with an average incidence rate of 15.89/100,000 inhabitants.

In the Americas, most of the CL endemic countries use passive surveillance as a routine reporting system; while active surveillance is mainly conducted during outbreaks or to assist specific inhabitants living in risk areas and of difficult access [15–18]. This study shows the importance of maintenance of adequate strategies for passive surveillance of CL and the need of a system that combines the information from different countries of the Americas, in order to investigate epidemiological features such as time and geographical distribution.

Our findings indicate a 44% increase in the number of CL cases, during the first five years of investigation, also reflected in the incidence rate. This growth could be attributed to an increase of reported cases from the Andean and Central America countries. The reasons for this increase are unknown, but could be explained by a variety of factors, including a true change in incidence or the improvement of clinical and laboratory diagnosis, and enhancement of the CL surveillance system in the endemic countries [15–19] (e.g. Argentina, Brazil, Colombia, Honduras, Paraguay, and Peru [20–23]). Then again, this increase might be even greater if we consider underreporting, which could arise due to lack of access to health services by patients, lack of awareness of the leishmaniasis reporting system by the staff, and the absence of clinical diagnosis, since subclinical or benign forms that cure spontaneously can occur [2, 3, 7, 8, 17, 19].

Furthermore, during the period of investigation, the Region faced several risk factors that contributed to environment and demographic changes (e.g., deforestation, modifications in the land use, increased migration, urbanization, climate change, etc.) that might have led to an increase of contact of human with vectors, therefore affecting the CL case trend [4, 20–29]. Studies conducted in Argentina, Bolivia, Brazil, Colombia, Mexico, and Peru have shown that frequency of leishmaniasis cases are often associated to climate changes and their impacts on the environment and economic activities, including agriculture [4, 19–30]. In Colombia, the Chaparral County reported an outbreak that lasted 5 years (2003–2008) and was considered the largest CL outbreak ever recorded in the country [21]. Also, Argentina recorded outbreaks in the Bella Vista, Formosa, and Salta Counties [31, 32]. In addition, the El Niño-Southern Oscillation (ENSO) has caused a growth of deforestation and of the Phlebotomine population, which has led to the rise of occurrence of CL [33–36]. Studies conducted in Colombia showed an association between increase of CL incidence rates during the El Niño and decline during the La Niña [4, 23]. On the contrary, in Bolivia the CL incidence rates increased during the La Niña and declined during the El Niño [37]. Therefore, climate forecast might be used to predict the leishmaniasis transmission risk in endemic countries affected by both phenomena [38].

The study also shows a different geographical distribution when applied to different countries and subnational levels, illustrating how limiting the use of each indicator can be when higher-risk areas need to be identified to prioritize actions. For example, Brazil has the highest number of cases reported, and at the same time one of the lowest incidence rates due to the size of the population, which emphasizes the need of new analysis strategies, including composite indicators such as the one used in this study. Moreover, it is important to use, when available, complementary information on the vectors, parasites, environment and social characteristic in order to understand the dynamic and traits of this disease [39].

The exploratory analytical tools used in this study to investigate the spatial and temporal distribution of CL in the Americas helped enhance knowledge of the epidemiology of CL and added value to the data reported by the countries. These exploratory methods and their graphic representations are an essential part for the understanding of the complexity of the data correlated over time [40]. This is particularly true in this study where our “sample” is the whole population under surveillance, and for more practical purposes it could be assumed that cases represented approximately the occurrence of CL in the population; in which statistical inference is not as useful as the descriptive method [41].

This study demonstrates that it is possible to provide, retrospectively, an overall picture of the epidemiological patterns of the endemic countries using secondary data, underlining the importance of passive surveillance as a key tool for management of the national control program and the use of the data analysis results for evidence-based programmatic actions and policy formulation. Our results should be interpreted in light of potential limitations posed by the use of data collected under passive surveillance, as showed and discussed in others studies [7, 8, 15–18]. The differences between reporting systems of different countries might affect the comparability of data. The countries included in this study have different types of denominator to estimate the CL incidence rate. Some countries apply the total population, others only the population within rural and urban areas, or only rural counties with CL transmission. The fact that this study uses a standard population as the denominator allowed the assessment and comparison of the incidence rate of CL between the 14 countries included. The results, however, may differ from data available in the countries or from other sources.

The results presented here have shown important advances in the implementation of leishmaniasis surveillance system in the Region, globally demonstrating the epidemiological status of CL in the Americas, but also supporting managers and international organizations in defining, directing and strengthening the actions of surveillance and control of leishmaniasis, in order to reduce cases, deformities and deaths caused by the disease.

Improvement of the surveillance system will allow a better understanding of the ongoing and changing dynamics of the CL transmission. Further operational research is required to design and implement preventive and control strategies [39]. For instance, operational research to determine the advantages in using multiple combined indicators, such as the two indicators used in this study, and to asses complementary indicators (e.g. biological, environmental, social, and other indicators for each subnational administrative entity) in order to generate a more detailed picture of the occurrence of leishmaniasis in the Americas [39, 42]. In addition, a more detailed and integrated analysis of the indicators along with geographical data, disaggregated at the second (or lower) subnational administrative level (counties), would also help to further clarify the epidemiological status of CL at the regional and country level.

Overall, surveillance activities should also be strengthened to provide disaggregated leishmaniasis data to the countries, ideally at the local level. It is also recommended the characterization of smaller territorial units according to their own unique human and physiographic traits (climatological, geomorphological, hydrographical, etc.), which can be highly complex and dynamic, and have the capacity to establish and/or influence an epidemiological situation. Generally, the characteristics of the area (concerning CL occurrence) are the result of interaction among different social groups that share the same space but may have different lifestyles, economies and environment [9, 20, 42].

In conclusion, this investigation indicates changes in the epidemiological patterns of CL in the American Region for unknown reasons, which could be due to changes in the risk factors as well as improvement of the diagnosis and notification. Further operational research and improvement of the surveillance system is needed, combined with activities to strengthen the capacity of the program. The joint use of these actions would lead to a culture of evidence–based in the leishmaniasis program management.
